# Evaluation of current genetic testing reports in German-speaking countries with regard to secondary use and future electronic implementation

**DOI:** 10.1038/s41431-020-0586-z

**Published:** 2020-02-13

**Authors:** Teja Falk Radke, Simon J. Patton, Elisabeth Pantazoglou, Julian Sass, Sylvia Thun

**Affiliations:** 1 Competence Center eHealth, Faculty of Healthcare, University of Applied Sciences Niederrhein, Krefeld, Germany; 2 European Molecular Genetics Quality Network (EMQN), Manchester Centre for Genomic Medicine, St Mary’s Hospital, Manchester, UK; 3 Berlin Institute of Health, Berlin, Germany

**Keywords:** Genetics research, Cancer genetics

## Abstract

Modern diagnostic methods (next-generation sequencing) are one of the current hopes with regard to a personalised medicine. By applying detailed genetic analysis, it is possible to not only improve the prediction of potential risks (as, e.g., concerning hereditary breast cancer) but also the precision of therapy by targeting it to a specific genetic variant. However, there is no international standard for creating, structuring and/or transferring the results of a genetic test report. This type of test report often contains large amounts of complex information, and a standardised and consistent structure would offer potential benefits to all. These include reduced expenditure of time (due to the elimination of information-conversion steps), improved safety (due to a reduction in the occurrence of transmission errors, misunderstanding or misinterpretation of content) and improved clinical information gathering (by the respective linkage to scientific data and literature). Especially in regard to secondary use, a standardised (electronic) format would improve the suitability of these data in retrospective studies and basic research. In this study, we analysed the format and content of 96 genetic testing reports (germline and somatic) from Germany, Switzerland and Austria. Based on these results, we summarised and discussed potentially critical data that were demonstrated to be reported inconsistently, and propose a baseline structure for reporting that would also ease future electronic conversion.

## Introduction

Commonly referred to in the context of “personalised medicine” or “precision medicine”, modern diagnostic technologies offer a powerful means for scientists and clinicians to choose an appropriate therapy, predict disease development or estimate hereditary risks. Whilst the overall concept of personalising treatment (choosing treatment on an individual level for each patient) is not new, it gained new momentum when the human genome was sequenced for the first time by the Human Genome Project (HGP) consortium [1]. Comparing the patient’s DNA directly to an established, validated reference sequence opened up new possibilities, not only for human geneticists, which focused on germline-associated (and thus inheritable) diseases, but also for molecular pathologists allowing them to establish a deeper insight into the mechanisms underlying somatic aberrations in tumours.

It took 20 international institutions in the HGP about 13 years (1990–2003) to sequence a single human genome at a cost of ~$2.7 billion, but since then time requirement as well as costs have dropped significantly due to enormous technical advances. As of now, even an individual genome can be analysed within a couple of days for less than $5000 using next-generation sequencing (NGS) [2] but, more importantly, NGS is also already well-established as an alternative to Sanger sequencing for small-scale targeted testing [3]. Also, while for specific tests, other established testing methodologies, such as fluorescence in situ hybridisation or real-time quantitative polymerase chain reaction, are currently still more cost-effective [4], the cost gap is decreasing rapidly, and it is predicted that NGS is likely to become the de facto standard for genetic testing.

However, having access to large amounts of detailed data also requires appropriate methodologies for handling and using them. Specifically, this can be considered as three distinct components:

1. *Evaluation.* Assessment of the test result quality requires that they are presented in a standardised format (i.e., that an unambiguous nomenclature is used), and that details of the materials and methods used are provided with their respective sensitivities, specificities and limitations.
2. *Interpretation*. Conclusions drawn from the results, e.g., disease risk estimation, are based on published literature, databases and predictions made in silico using computational algorithms. Interpretation of genetic testing results can be challenging [5], especially for novel unclassified variants. Therefore, the resources used to form an interpretative conclusion should be clearly summarised on a genetic test report.
3. *Exchange*. Especially in regard to electronic reporting of patient tests, the results must always be communicated to the referring physician in a secure format. As this information is also transmitted frequently across national (country) boundaries, especially in rare disease when an external expert opinion may be required, it is important that a standardised format (template) is used to ensure that the correct information is communicated to the end user.

While recommendations and guidelines on reporting do exist, these do in general focus on the clinical unambiguity of the results that are reported back to the referring clinician [6–8] rather than a potential secondary use. Nevertheless, as research and development of new therapies and drugs are always based on access to a sufficient amount of valid, unambiguous data, this aspect should not be underestimated. This is especially true for fast-evolving domains, such as genetic testing. Therefore, the “GENeALYSE” project (funded by the Ministry of Culture and Science of the State of North Rhine-Westphalia, Germany) aimed at identifying the elements of a genetic test report that should be included in a future specification (implementation guide) for electronic reporting, with additionally placing special emphasis on potential key data for secondary use. For this purpose, the authors analysed the format and content of genetic testing reports from various German-speaking laboratories, all of which had participated in external quality assessment (EQA) activities organised by the European Molecular Genetics Quality Network (EMQN).

The presented work reports the results from this study, and discusses further aspects potentially relevant to secondary use and future electronic reporting, indicating a need for standardised structure and content in genetic testing reports.

## Materials and methods

### Reports used in the study

Ninety-six genetic test reports were provided for analysis by the EMQN. The reports represented the output from 63 distinct laboratories from three different German-speaking countries (Germany, Switzerland and Austria), participating in eight different EQA schemes (Table 1). For assessment, reports that focussed on germline variants and used peripheral blood as a source for DNA were distinguished from reports that analysed somatic variants in samples derived from formalin-fixed paraffin embedded (FFPE) tumour tissue, and were categorised as “human genetics” or “molecular pathology”, respectively. The only exception was the EQA scheme for *BRCA* gene testing in ovarian cancer, which used tumour tissue, and subsequently was categorised as belonging to the area of molecular pathology, albeit focussing on a germline variant.

**Table 1:** Overview on reports used from ring trials.

Trials	Reports (total)	Unique laboratories
Human genetics	71	56
Hereditary breast cancer (*BRCA1*)	37	
Huntington disease (*HTT*)	18	
Haemochromatosis (*HFE*)	16	
Molecular pathology	25	12
Colorectal cancer adenocarcinoma (*KRAS, NRAS* and *BRAF*)	5	
Lung adenocarcinoma (*EGFR*)	5	
Skin melanoma (*BRAF*)	5	
Ovarian cancer (germline) (*BRCA1* and *BRCA2*)	4	
Ovarian cancer (somatic) (*BRCA1* and *BRCA2*)	8	
Total	96	63^a^

^a^Five of the laboratories participated in ring trials for human genetics, as well as in trials for molecular pathology.

All of the reports analyses were anonymised by the EMQN and identified only by a unique code. This code was identical for each laboratory, independent of the EQA scheme, and therefore enables tracking of the laboratory between each EQA scheme. All the study team members consented to a non-disclosure agreement.

Using outputs from the EQA schemes facilitated the direct comparison of reports submitted for a distinct clinical indication. In addition, since the original data (mock clinical referral including patient information) were available (provided by the EMQN to accompany the samples), it was possible to retrospectively assess the presence or absence of key pieces of information against a scoring matrix of key requirements.

### Report order evaluation and identification of key elements

For each report, page count, as well as the structural order, of the following sections was assessed:

1. Indication and patient data (including original test request information, clinical referral and sample information).
2. Materials and methods.
3. Results.
4. Interpretation and expert opinion.
5. Details/additional data (if applicable).

In order to identify “key elements”, all reports then were analysed in detail and broken down into their basic components (such as “patient first name”, “sample source”, “official gene name”, etc.), resulting in a list of information that in an ideal case, should be included in any paper-form report. This list then was reviewed with clinical experts and expanded to include additional elements that are currently not included in genetic testing reports by default, but which would be beneficial with regard to electronic processing. For example, most reports currently do include the date the analysis was requested and the date the report was written, but do not always or rarely state when the sample was received, processed or when the analysis was finished, respectively. While usually not of much importance, these data may become relevant in the circumstances of a suspicious result or other deviations from expectations, and could be easily integrated automatically into an electronic form without burdening the user with additional work. In addition, this list was analysed for compliance with the ISO 15189 (Medical laboratories—Requirements; third revision 2012) [9], since this standard also states elements that should be mandatory in laboratory documentation and reporting, respectively.

Finally, all reports then were analysed for each single key reporting element, resulting in a statistical evaluation regarding its presence/absence within each report.

## Results

### Overall structure

In general, a strong variation already in the sheer amount of text in between the respective reports was observable, as the length of the reports varied from minimalistic (a single page, text only) to extensive (up to seven pages, including multiple tables and/or scanned PCR results).

Of course, as represented in Table 2, average page count correlates with the complexity of the respective clinical question and/or applied tests. In detail, reports on colorectal cancer adenocarcinoma mostly consisted of two or more pages, while reports on haemochromatosis or ovarian cancer never exceeded two pages. The varying amount of pages within each trial, however, can be correlated to the different report structures (i.e., templates) used by individual laboratories, as for example, each report of one specific laboratory consisted of at least six pages in molecular pathology trials.

**Table 2:** Overview on pages per report and trial.

Pages/ report	Human genetics			Molecular pathology				
	Hereditary breast cancer	Huntington disease	Haemochromatosis	Colorectal cancer adenocarcinoma	Lung adenocarcinoma	Skin melanoma	Ovarian cancer (germline)	Ovarian cancer (somatic)
1	14 (37.8%)	16 (88.9%)	11 (68.8%)	1 (20.0%)	3 (60.0%)	1 (33.3%)	3 (75.0%)	5 (62.5%)
2	20 (54.1%)	1 (5.6%)	5 (31.3%)	2 (40.0%)	1 (20.0%)	1 (33.3%)	1 (25.0%)	2 (37.5%)
3+	4 (8.1%)	1 (5.6%)	1 (0.0%)	2 (40.0%)	1 (20.0%)	1 (33.3%)	0 (0.0%)	0 (0.0%)
Total	37	18	16	5	5	3	4	8

The results are given as percentage of the respective total of reports within the trial (stated in brackets below; total percentage may vary due to rounding).

Regarding the order, as depicted in Table 3, half of the 63 reports (32, or 50.8%) were analogous to the scientific IMRaD standard (introduction, materials and methods, results and discussion) with indication and patient data first, materials and methods second, results third, interpretation and recommendations fourth and, if applicable, additional data fifth. The remaining reports mostly varied in the position of materials and methods only, positioned either after interpretation (20, or 31.7%) or after results (5, or 7.9%). However, reports from six laboratories (9.5%) were structured completely differently, omitting, splitting up or merging relevant sections, respectively.

**Table 3:** Overview on the order of analysed reports.

Order	Reports^a^
Indication/materials and methods/results/interpretation/additional data	32 (50.8%)
Indication/results/interpretation/materials and methods/additional data	20 (31.7%)
Indication/results/materials and methods/interpretation/additional data	5 (7.9%)
Other	6 (9.5%)

Reports from 63 unique laboratories were analysed for their respective order of the sections containing indication/patient data, materials and methods, results, interpretation/recommendations and further data.

^a^Total percentage at 99.9% due to rounding.

### Key elements and quantitative evaluation

Following the analysis of the reports provided, a set of key elements were determined and grouped (Table 4), with 1–5 identical to those mentioned in the structural analysis. Key elements not belonging to Groups 1–5 were categorised as “Other/Formal/Legal”. In case of variant description (gene and protein level), it was also evaluated if data were presented in accordance to the terminology proposed by the Human Genome Variation Society (HGVS). Similarly, statements on estimated pathogenicity were expected to adhere to the five-tier classification system recommended by the American College of Medical Genetics and Genomics.

**Table 4:** Overview of key element groups.

1. Patient and sample information	
Patient first name	Family disease history
Patient last name	Sample material
Patient sex	Sample form/solvent/additives
Patient date of birth	Sample source
Patient family members	Sample external ID
2. Indication/request	
Suspected disease	Previous/other testing
Anamnesis	Original request/gene(s) to be tested
3. Materials and methods	
Range of genes tested/panel used	Method/technical limitations
Methods applied	Primers/library used
Devices used (manufacturer/model)	Sensitivity/detection limit
Reference sequence used (NM_/LRG)	Analysis details (IVS; read depth)
4. Results	
Variant(s) (not) detected/confirmed	Zygosity [g] or percentage [s]
Variant description, gene (HGVS)	Trivial/traditional name
Variant description, protein (HGVS)	
5. Interpretation and expert opinion	
Analysis software used (in silico) [g]	Mutation consequences/mechanism details
Databases used	Risk for patient
Evaluation pathogenic/disease associated [g]	Hereditary risk explicitly mentioned [g]
Estimated pathogenicity class value (ACMG) [g]	Disease/Variant literature reference(s)
6. Other/formal/legal/recommendations	
Patient consent given	Storage/disposal of material after testing
Genetic counselling performed	Pages numbered (x of y)
Counselling of family members recommended [g]	Pages with patient’s name if more than one page
Genetic counselling along with results [g]	Date request/sample received
Recommendations (therapy/further testing)	Date analysis started
Confirmative testing done/required/recommended	Date report written

Key elements identified in the report analysis were categorised into six distinct groups. Note that this does not reflect order of appearance or position in the analysed reports, but was solely used as a checklist for the subsequent content analysis. [g]/[s]: applicable to genomic or somatic testing, respectively.

It should be noted that not all of the key elements are considered mandatory in every report. For example, data on the patient’s family, hereditary risk and genetic testing/counselling of family members may be irrelevant without a germline-associated background. However, it would be beneficial to include these data into the report for the sake of completeness in a secondary use setting (e.g., research).

All reports were analysed for these key elements, and the respective level of detail was assessed (rated as “Complete”, “Partial, unclear or misleading within context” or “Missing”, respectively). The respective results are presented in Tables 5–10 (if not stated otherwise, the results are given as total number and percentage of reports from all unique laboratories, 63 in total).

**Table 5:** Key element Group 1 (patient and sample information).

1. Patient and sample information	Reports	% complete	% partial	% missing
Patient first name	63	63 (100.0%)	0 (0.0%)	0 (0.0%)
Patient last name	63	63 (100.0%)	0 (0.0%)	0 (0.0%)
Patient sex	63	52 (82.5%)	0 (0.0%)	11 (17.5%)
Patient date of birth	63	63 (100.0%)	0 (0.0%)	0 (0.0%)
Patient family members^a^	37	12 (32.4%)	2 (5.4%)	23 (62.2%)
Family disease history^a^	56	34 (60.7%)	7 (12.5%)	15 (26.8%)
Sample material	63	53 (84.1%)	4 (6.3%)	6 (9.5%)
Sample form/solvent/additives^b^	63	11 (17.5%)	1 (1.6%)	51 (81.0%)
Sample source	63	16 (25.4%)	4 (6.3%)	43 (68.3%)
Sample external ID	63	49 (77.8%)	5 (7.9%)	9 (14.3%)

^a^Only taken into account if provided with initial request.

^b^Two laboratories reported the sample as “lyophilised” although all laboratories received DNA in TE buffer.

**Table 6:** Key element Group 2 (indication/request).

2. Indication/request	Reports	% complete	% partial	% missing
Suspected disease/purpose^a^	9	8 (88.9%)	0 (0.0%)	1 (11.1%)
Anamnesis	63	44 (69.8%)	12 (19.0%)	7 (11.1%)
Previous/other testing^a^	37	9 (24.3%)	9 (24.3%)	19 (51.4%)
Original request/gene(s) to be tested	63	25 (39.7%)	18 (28.6%)	20 (31.7%)

^a^Only taken into account if clearly provided with initial request.

**Table 7:** Key element Group 3 (materials and methods).

3. Materials and methods	Reports	% complete	% partial	% missing
Range of genes tested/panel used^a^	63	57 (90.5%)	5 (7.9%)	1 (1.6%)
Methods applied	63	52 (82.5%)	11 (17.5%)	0 (0.0%)
Devices used (manufacturer/model)	63	21 (33.3%)	8 (12.7%)	34 (54.0%)
Reference sequence used	63	52 (82.5%)	4 (6.3%)	7 (11.1%)
Method/technical limitations^b^	53	19 (35.8%)	0 (0.0%)	34 (64.2%)
Primers/library used	63	16 (25.4%)	13 (20.6%)	34 (54.0%)
Sensitivity/detection limit	63	24 (38.1%)	7 (11.1%)	34 (50.8%)
Analysis details (IVS; read depth)^c^	47	10 (21.3%)	13 (27.7%)	24 (51.1%)

^a^One laboratory, without explanation, only analysed one of the two genes requested.

^b^Not taken into account for CAG-repeat tests (ten reports).

^c^Not taken into account if specialised methods/kits were used.

**Table 8:** Key element Group 4 (results).

4. Results	Reports	% complete	% partial	% missing
Variant(s) (not) detected/confirmed^a^	63	61 (96.8%)	2 (3.2%)	0 (0.0%)
Variant description, gene (HGVS)^b^	51	51 (100.0%)	0 (0.0%)	0 (0.0%)
Variant description, protein (HGVS)^b^	51	50 (98.0%)	1 (2.0%)	0 (0.0%)
Zygosity [g] or percentage [s]^c^	61	58 (95.1%)	0 (0.0%)	3 (4.9%)

[g] germline, [s] somatic analysis.

^a^Among all (96): three cases were of contradicting reports, five were unclear if “no pathogenic mutation found” was the same as “no mutation found”.

^b^Not taken into account for CAG-repeat tests (ten reports) as well as for two negative reports.

^c^Not taken into account for two negative reports.

**Table 9:** Key element Group 5 (interpretation and expert opinion).

5. Interpretation and expert opinion	Reports	% complete	% partial	% missing
Analysis software used (in silico) [g]^a^	37	20 (54.1%)	3 (8.1%)	14 (37.8%)
Databases used^a^	37	29 (78.4%)	7 (18.9%)	1 (2.7%)
Evaluation pathogenic/disease associated [g]^b^	47	32 (68.1%)	7 (14.9%)	8 (17.0%)
Estimated pathogenicity class value (IARC) [g]^a^	37	20 (54.1%)	15 (40.5%)	2 (5.4%)
Variant consequences/mechanism details	63	47 (74.6%)	1 (1.6%)	15 (23.8%)
Risk for patient^c^	57	42 (73.7%)	2 (3.5%)	13 (22.8%)
Hereditary risk explicitly mentioned [g]	56	28 (50.0%)	4 (7.1%)	24 (42.9%)
Disease/variant literature reference(s)^d^	61	27 (44.3%)	2 (3.3%)	32 (52.5%)

[g] germline, [s] somatic analysis.

^a^Only applicable to one trial (BRCA1 w. unknown mutation).

^b^Not taken into account for one trial (HFE; nine negative reports).

^c^Applicable for germline trials (56 reports) plus one positive molecular pathology report.

^d^Not taken into account for two negative reports.

**Table 10:** Key element Group 6 (other/formal/legal/recommendations).

6. Other/formal/legal/recommendations	Reports	% complete	% partial	% missing
Patient consent given	63	5 (7.9%)	2 (3.2%)	56 (88.9%)
Genetic counselling performed	63	6 (9.5%)	1 (1.6%)	56 (88.9%)
Counselling of family members recommended [g]	56	40 (71.4%)	4 (7.1%)	12 (21.4%)
Genetic counselling along with results [g]	55	48 (87.3%)	2 (3.6%)	5 (9.1%)
Recommendations (therapy/further testing)	63	46 (73.0%)	5 (7.9%)	12 (19.0%)
Confirmative testing done/required/recommended	63	21 (33.3%)	0 (0.0%)	42 (66.7%)
Storage/disposal of material after testing	63	2 (3.2%)	0 (0.0%)	61 (96.8%)
Pages numbered (x of y)	63	40 (63.5%)	4 (6.3%)	19 (30.2%)
Patient’s name on each page if >1 page	30	19 (63.3%)	0 (0.0%)	11 (36.7%)
Date sample drawn^a^	63	0 (0.0%)	0 (0.0%)	63 (100.0%)
Date request/sample received	63	57 (90.5%)	2 (3.2%)	4 (6.3%)
Date analysis started^a^	63	0 (0.0%)	0 (0.0%)	63 (100.0%)
Date report written	63	59 (93.7%)	0 (0.0%)	4 (6.3%)

[g] germline, [s] somatic analysis.

^a^Key elements not derived from report analysis, added with regard to a potential retrospective analysis.

## Discussion

### Recommended structure for easier accessibility

Whilst all the reports evaluated contained the basic essential information needed by the requesting clinician (which, in general, is basically the result of the analysis and related implications), the analysis also showed that the general reporting structure and content order varies considerably. This might be only an inconvenience when handling an individual case, where missing data might be more easily demanded, but may cause problems when evaluating multiple reports from different laboratories. Especially in larger studies, this could make the analysis significantly more time consuming, and increase the risk of data being missed or misinterpreted.

Therefore, especially in regard with secondary use in retrospective studies, we suggest that a more formalised structural requirement for reports could be inspired by the scientific IMRaD standard mentioned above. Although laboratories should be allowed to have a certain degree of flexibility, especially with regard to the order of the specific elements, we suggest that reports should be subdivided/grouped accordingly:

Indication: data on patient, sample and clinical referral indication.

Materials/methods: methodological approaches, devices and panels used (including associated limitations, sensitivity and specificity), as well as databases consulted.

Results: description of variant(s) detected (or excluded) using HGVS nomenclature.

Assessment: biological and clinical interpretation of results including estimated pathogenicity, disease risk assessment and (if applicable) recommendations.

Details: detailed results, further information on disease/variant, external references, etc.

Using this adapted “IMRAD” grouping (introduction, materials and methods, results, assessment and discussion) would facilitate better data sharing, especially if adhering to established electronic data standards, as it would allow the user to filter/fetch the information needed selectively. Note that, while most of the 63 laboratories in this study were already compliant to this structuring, reports from six laboratories still demonstrated structures that did not conform to it and subsequently made analysis more difficult.

### Missing key elements may cause misinterpretation or hamper secondary use

Among the pieces of key information analysed within the study, as demonstrated in the results, many were not given consistently in the reports. While these may be of no consequence with regard to the individual medical case, those data might be crucial for use in clinical studies, help in troubleshooting, ensure legal protection, etc. This includes but is not limited to the following examples:

1. Gender and referral information: most prominent and potentially critical is the lack of the patient’s gender in 11 of the 63 reports (17.5 %). Even though the patient is referred to partially as “Ms/Mrs” or “Mr” this does not necessarily mean that the patient is biologically female or male. This information is often essential for subsequent risk assessment (e.g., in the case of specific variants, such as in the *BRCA* genes). In addition, only 34 of the 56 laboratories (60.7%) fully restated the clinical referral details on the patient’s family history as they were provided by the EMQN, although this value varied in between trials. As this classification would only cover for a normal karyotype, known aberrations (either gonosomal or autosomal) should be clearly indicated in the indication section. Also, note that laboratories in general do not perform a confirmation of the biological sex as long as it is not part of the requested testing or part of internal quality control.
2. Incomplete/missing data: reports often did not reflect all parts of the clinical referral information originally provided with the request. For example, in hereditary breast cancer (*BRCA* genes) scheme, the female patient’s family history consisted of four pieces of information: the patient was an only child, with no children herself, there was no history of disease within the family and the paternal branch of the family tree was mostly males. Among the 37 participating laboratories in this EQA, only 11 (29.7%) included all this information, 14 (37.8%) partially, while the remaining 12 (32.4%) did not include any of these details. Furthermore, in the same EQA, only 9 laboratories fully, plus 9 laboratories partially (each 24.3%) stated that previous tests (including results) had been undertaken, whilst 19 laboratories (51.4%) omitted this information.
3. Materials and methods: a large proportion of the reports lacked detailed technical information that might be of value not only in broader retrospective studies, but also in evaluation of quality of the results. In particular, sensitivity, specificity and other limitations of method or kit applied should be clearly stated.
4. Results and assessment: one of the EQA’s (*BRCA* gene testing in ovarian cancer in the context of PARP-inhibitor therapeutic recommendation) demonstrated the importance of context-specific presentation and phrasing of results. Of the eight laboratories participating, 2 (25.0%) stated “no mutation found”, 3 (37.5%) “no pathogenic mutation found” and 3 (37.5%) “mutation c.736T>G found–likely benign/assumed neutral”. Postulating that due to future findings a variant originally considered as of uncertain significance or even benign might indeed become clinically relevant [10], re-analysis of reports would only be applicable to the last three. For the same reason, while it might be necessary to restrict the results to findings of interest only (i.e., when using larger panels), all other findings (i.e., non-pathogenic variations) should still be available in form of a summary or as additional data (e.g., as a version controlled supplementary download). Also, to avoid confusion whether “mutation” refers to simply “a change” or to “a disease-inducing change”, the neutral term “variant” should be used instead. Similarly, when using the term “pathogenic”, it has to be made clear whether this means “definitely disease causing” or “disease causing under specific conditions”. In the latter case, these conditions/mechanisms should then also be clearly stated (e.g., dominant, recessive and X-linked).
5. Patient’s informed consent given: while 56 of the 63 (88.9%) laboratories did not state anything on the informed consent this can be seen as an example for an information that is taken for granted (as it is required by law in the countries involved in this study) and therefore omitted. However, also for legal protection, the laboratories should always state that they verified whether the patient’s informed consent was given.
6. Sample handling after testing: only 2 of the 63 laboratories (3.2%) clearly stated that the sample material was either stored or discarded following analysis. Again, this is likely because, in general, samples are retained after testing and therefore this is taken as granted if not stated otherwise. However, as current development tends towards patients managing or at least having access to all their (electronic) health files, they might assume that the sample is simply used up during testing or discarded afterwards, rather than stored. Therefore, this statement is important not only in case of a potentially required re-testing, but also with regard to patient empowerment.

Thus, if laboratories consider potential secondary use in their reports, as well as making their reports easily convertible into an electronic format, the elements listed in this study may offer an insight on key requirements for inclusion in a report. In brief, the overall information should be broken down into single pieces of data, which then can be coded and retrieved unambiguously by application of established terminologies and standards. However, care must be taken not to omit aspects that are often considered as obvious or taken as granted, for this might result in confusion, especially among non-experts (such as the patients themselves).

### Conclusions: secondary use would benefit from use of electronic and nomenclature standards within genetic testing reports

Our study clearly demonstrates that the clinical reports analysed, while of course conveying the necessary information relevant to the requesting clinician, and therefore meeting the needs of clinical decision-making, often lack details and structuring that would make the report useable as a stand-alone source for secondary use or direct electronic conversion, respectively. However, the landscape of genetic testing is changing rapidly and new targeted therapies are quickly coming to market. To facilitate this drug companies are dependent on running informative clinical trials and effective stratification of patient cohorts is essential in this activity. Therefore, we argue that the informativeness of clinical reports could be improved through better standardisation of structure and content, which would be beneficial for clinical studies, and electronic communication of results.

A uniform structure would reduce the risk of misinterpretation and loss of information at a national and global scale. Here, internationally accepted standards and terminologies are key elements in order to ensure technical interoperability and clinical unambiguity. In cases where there are multiple applicable and established terminologies to choose from (e.g., Orphanet, the Human Phenotype Ontology or PhenoTips for clinical descriptions), laboratories should be free to select their own discretion as long as it is clearly stated.

The authors suggest that the use of HL7’s formats (either Clinical Document Architecture or Fast Healthcare Interoperability Resources), in combination with the international terminologies SNOMED Clinical Terms (Systematized Nomenclature of Medicine—Clinical Terms; SNOMED international; www.snomed.org) and Logical Observation Identifiers Names and Codes (Regenstrief Institute; loinc.org) in an electronic genetic testing report could fulfil these requirements. Appliance of these terminologies is expected to not only already cover all the elements elaborated in this study but also future aspects, since both are under continuous development.

### Limitations of this study

This study was performed on reports derived from the EQA scheme organised by the EMQN; therefore, it can be argued whether they were probably not taken as serious as real tests (although this is a fundamental precondition for participation in the trials), or on the contrary, the laboratories took even more care over their EQA reports than routine clinical referrals. In addition, legislative regulations differ in between Germany, Austria and Switzerland, which might affect those aspects summarised in point 6 “Other/Formal/Legal/Recommendations”.

Finally, the laboratory setting with respect to test type may affect the presence of certain information on a report. For example, specific national regulations may exclude laboratories from adding therapeutic recommendations to clinical test reports.

### Outlook

Based on the findings presented in this study, and HL7’s specifications for genetic testing reports [11], it is planned to define an adapted and enhanced version as a proposal for German electronic genetic testing reports within the GENeALYSE project. This implementation guide will then be distributed free-of-charge to all respective stakeholders. Especially in regard to (electronic) exchange of data on a global scale, this might then be used as a basis for discussion for an international version.

However, it has to be kept in mind that national laws, especially with regard to data protection, must to be taken into consideration. Data that are collected routinely in one country might not be allowed to be disclosed, stored or even asked for in another country. An example would be “Ethnicity”, which is considered a common information in the USA, but highly problematic in Germany. In these cases, an entry should be provided and marked as “unavailable” or similar to indicate that it has been omitted intentionally.
